# Redundant Vasodilator Pathways Underlying Radial Artery Flow-Mediated Dilation Are Preserved in Healthy Aging

**DOI:** 10.1155/2014/876125

**Published:** 2014-05-21

**Authors:** Kevin D. Ballard, Michael E. Tschakovsky, Amanda L. Zaleski, Donna M. Polk, Paul D. Thompson, Francis J. Kiernan, Beth A. Parker

**Affiliations:** ^1^Division of Cardiology, Hartford Hospital, 80 Seymour Street, Hartford, CT 06102, USA; ^2^School of Kinesiology and Health Studies, Queen's University, 28 Division Street, Kingston, ON, Canada K7L 3N6; ^3^Department of Health Sciences, University of Hartford, 200 Bloomfield Avenue, West Hartford, CT 06117, USA

## Abstract

*Background.* Blocking nitric oxide (NO) and vasodilator prostanoids (PN) does not consistently reduce flow-mediated dilation (FMD) in young adults. The impact of aging on the contribution of NO and PG to FMD is unknown. *Methods.* FMD was measured in older adults (*n* = 10, 65 ± 3 y) after arterial infusion of saline, N(G)-monomethyl-L-arginine (L-NMMA), and ketorolac + L-NMMA. Data were compared to published data in young adults. *Results.* L-NMMA reduced FMD in older adults (8.9 ± 3.6 to 5.9 ± 3.7%) although this was not statistically significant (*P* = 0.08) and did not differ (*P* = 0.74) from the reduction observed in young adults (10.0 ± 3.8 to 7.6 ± 4.7%; *P* = 0.03). Blocking PN did not affect FMD in young or older adults. In older adults, L-NMMA reduced (*n* = 6; range = 36–123% decrease), augmented (*n* = 3; 10–122% increase), or did not change FMD (*n* = 1; 0.4% increase). After PN blockade, FMD responses were reduced (*n* = 2), augmented (*n* = 6), or unaffected (*n* = 1). *Conclusions.* NO or PN blockade did not consistently reduce FMD in healthy older adults, suggesting the existence of redundant vasodilator phenotypes as observed previously in young adults.

## 1. Introduction

Conduit artery flow-mediated dilation (FMD) is a widely used noninvasive method to assess endothelial function. Diminished FMD is widely assumed to reflect impaired dilator function of nitric oxide (NO) [1], based on several studies reporting the absence or substantial attenuation of FMD following infusion of the NO synthase (NOS) inhibitor N(G)-monomethyl-L-arginine (L-NMMA) [2–6]. In support, a recent meta-analysis of 13 crossover studies utilizing L-NMMA infusion and temporary distal ischemia found a substantial contribution of NO (72%) to the conduit artery FMD response in healthy adults [7]. However, other studies failing to significantly alter FMD with L-NMMA [8–10] indicate potential involvement of additional vasodilators (such as vasodilator prostanoids (PN) [11] and endothelium-derived hyperpolarizing factor (EDHF) [12, 13]). Evidence of a concurrent or compensatory role of alternative dilators in health and disease [12, 14] suggests that these alternative vasodilators may be important for cardiovascular health. In support of this hypothesis, FMD following proximal cuff occlusion, which is only partially reduced by NO blockade [2], predicts future cardiovascular events as effectively as FMD following distal occlusion [15]. Consequently, interpreting conduit FMD as primarily mediated by NO release and endothelial dysfunction as a loss of the NO dilator system overlooks the relevance of redundant vasodilator systems for cardiovascular function and their potential as targets for therapies aimed at ameliorating cardiovascular risk with age and disease.

We recently investigated the mechanisms underlying radial artery (RA) FMD in young men and women, finding that blocking NO and PN reduced the vasodilator response to ischemia in some subjects but not in others [8]. For example, individual variability in the FMD response following L-NMMA and/or PN blockade by ketorolac was observed, and only half of the young subjects exhibited an observable (and fairly modest) effect of L-NMMA on RA FMD. As discussed above, these data suggest that variable and redundant dilator pathways contribute to conduit artery FMD in healthy young men and women. Whether these redundant vasodilator pathways are impacted by aging is unknown, as previous work investigating the contribution of NO and PN to rest and exercise limb hyperemia in aged humans has not been extended to FMD or has focused exclusively on the NO system [16–19]. Therefore, the present investigation extends our previous findings [8] by examining differences due to healthy aging on the vasodilator pathways underlying RA FMD, a model utilized in previous infusion studies [3, 4, 6, 9]. Specifically, we sought to determine whether aging impairs the FMD response that persists following single and combined NO and PN blockade [8]. We hypothesized that blocking NO and PN synthesis would more substantially reduce RA FMD in older adults, indicating impaired redundant vasodilator mechanisms with age that could deleteriously impact cardiovascular health.

## 2. Methods

### 2.1. Subjects

We enrolled healthy, nonsmoking older adults (*n* = 10, 5 men and 5 women, ages 60–79). Data from young adults (*n* = 16, 8 men and 8 women, ages 20–35) have been reported previously [8] and were used for comparison purposes in the present study. Both young and older individuals were excluded if they met the criteria described previously [8]. All participants provided written, informed consent as approved by the Institutional Review Board at Hartford Hospital.

### 2.2. Testing Procedures

Detailed methods have been described previously [8] and are identical in the present investigation. Interested subjects reported to the laboratory following a 12 h fast in which data for inclusion/exclusion criteria [8] were assessed. Next, a venous blood draw was performed for the analysis of whole blood viscosity, blood lipid levels, and hemoglobin (Clinical Laboratory Partners, Hartford, CT, USA) and resting blood pressure, heart rate, and anthropometrics were measured. On a separate day, enrolled participants reported to the laboratory for an invasive catheterization visit following a ≥8 h fast and abstinence from exercise, pain medications, and herbal supplements for ≥24 h.

### 2.3. Catheterization Visit

Detailed methods for the local infusion of study drugs have been described previously [8]. Briefly, to locally infuse saline and study drugs, a 20-gauge Teflon catheter was inserted into the left brachial artery at the level of the antecubital fossa following anesthetization of the overlying skin. A three-port connector in series with a catheter-transducer system was used to administer saline and study drugs via the arterial catheter. One port was used to measure arterial pressure while the two remaining ports allowed for drug infusions and continuous saline administration for flushing.

Following a 30 min supine rest period, saline was infused at 2 mL/min for 10 min to establish a control condition for any effects of infusate administration on FMD. Radial artery FMD was then performed. In brief, the RA was imaged ~10 cm distal to the antecubital fossa using a 5–12 MHz multifrequency linear-array transducer attached to a high-resolution ultrasound machine (Terason t3000; TeraTech Corp., Burlington, MA, USA). Using the same ultrasound machine Doppler velocity was also continuously measured using a 60° angle of insonation which remained constant throughout the study. Resting RA diameter and velocity were recorded for 1 min before the inflation (300 mmHg for 5 min) of a pneumatic occlusion cuff placed around the participant's wrist. Diameter and velocity were recorded continuously beginning 30 s before cuff deflation until 3 min after deflation. After a 10 min rest period and infusion of L-NMMA (Clinalfa, Laeufelfingen, Switzerland) for 10 min at 5 mg/mL, 2 mL/min, RA FMD was repeated to determine the impact of NO blockade. Following another 10 min rest period, ketorolac (Toradol; Abbott Labs, Abbott Park, IL, USA) was infused for 5 min at 600 *μ*g/mL, 2 mL/min, immediately followed by a maintenance dose of L-NMMA for 5 min (5 mg/mL, 2 mL/min). RA FMD was then repeated a third time. Drug doses equaled or exceeded those used in previous studies successfully blocking NO and PN in the forearm [4, 6, 18, 20–22].

### 2.4. Diameter and Velocity Data Analysis

Radial artery FMD data were analyzed as reported previously [8]. Briefly, offline analysis of diameters and velocities was performed using Brachial Analyzer software (Medical Imaging Applications LLC, Coralville, IA, USA). Analyses were performed by a technician blinded to any subject information. Only end diastolic diameters, triggered by the corresponding Doppler waveform, were captured for diameter analyses. Radial artery FMD was expressed as percent change in dilation relative to baseline for each trial.

Mean velocity matched to the corresponding diameter was used to calculate shear stress in dyn/cm^2^ [4 *μ·*V/D; *μ* = blood viscosity (mPA*·*sec at 60 rpm);* D* = arterial diameter (cm);* V* = velocity (cm/sec)]. The postocclusion shear stress area under the curve (AUC SS) was calculated from cuff release until the time of peak diameter [23, 24] and used to normalize FMD [FMD/AUC SS]. Radial artery blood flow [RBF (mL/min) = blood velocity (cm/s)  ∗  *π*  ∗  (radial diameter (cm)/2)^2^  ∗  60] and mean intra-arterial blood pressure (MAP (mmHg)) measured at the baseline of each trial were used to calculate radial vascular conductance [RVC = RBF/MAP  ∗  100].

### 2.5. Statistical Methods

Statistical analyses were performed with SPSS 19.0. A one-way ANOVA was performed to compare baseline differences between age groups. Two-way ANOVA was used to analyze RA FMD responses with trial (drug infusion) as the within-subject (repeated measures) factor, group as the between-subject factor, and a group-by-trial interaction. Significance was determined at *P* ≤ 0.05 and a Tukey post hoc adjustment was applied to significant main effect and interaction comparisons. A chi square test was used to compare the number of young versus older adults who exhibited decreased RA FMD from saline to L-NMMA and then from L-NMMA to ketorolac + L-NMMA. Linear regression was performed to predict if the change in RA FMD from saline to L-NMMA was explained by select independent variables.

## 3. Results

### 3.1. Participant Characteristics

Characteristics for the young adults have been reported [8] and are summarized in Table 1 in comparison to older adults.

### 3.2. Baseline Hemodynamic Parameters across Infusion Trials

Means for resting RA diameter, RBF, MAP, and RVC between age groups measured immediately following infusions of saline, L-NMMA, and ketorolac + L-NMMA are shown in Table 2. In older adults, ketorolac + L-NMMA infusion increased (*P* < 0.01) MAP compared with saline. The lack of a significant trial ∗ group interaction (*P* = 0.68) indicates that the MAP response to combined infusion did not differ from the increase (*P* < 0.01) observed in young adults.

### 3.3. RA FMD Responses across Infusion Trials

**Figure 1:**
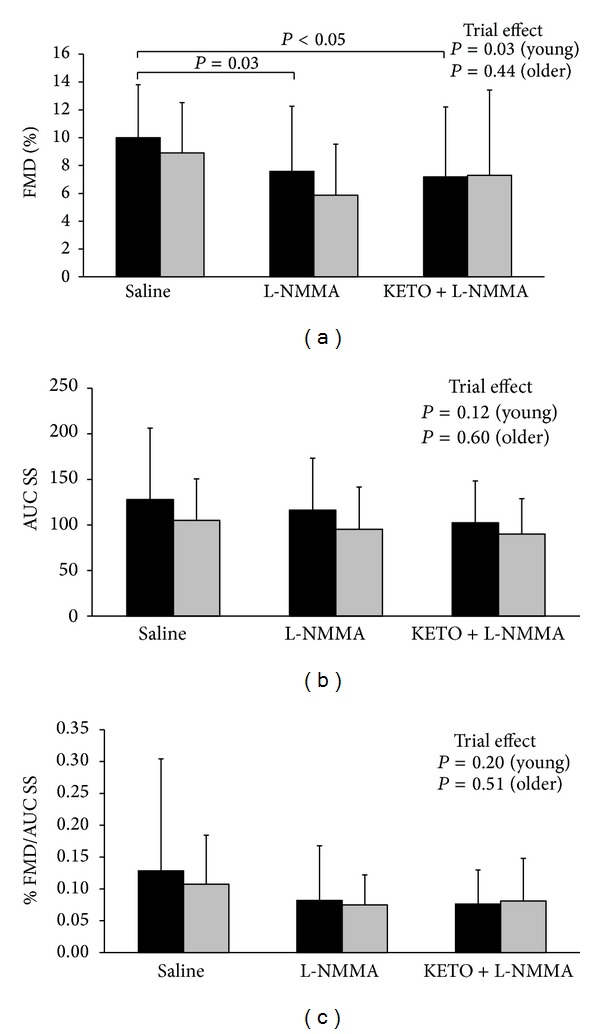
Group means ± SD for young (data from young participants have been published previously [8]) (*n* = 16; black bars) and older (*n* = 10; grey bars) adults are shown for FMD (a), AUC SS (b), and FMD/AUC SS (c) with *P* values for trial effects (within each group) and any significant differences between conditions on each graph. *n* = 9 older adults in the KETO + L-NMMA trial. AUC SS: postocclusion shear stress area under the curve; FMD: flow-mediated dilation; L-NMMA: N(G)-monomethyl-L-arginine; KETO: ketorolac.

**Figure 2:**
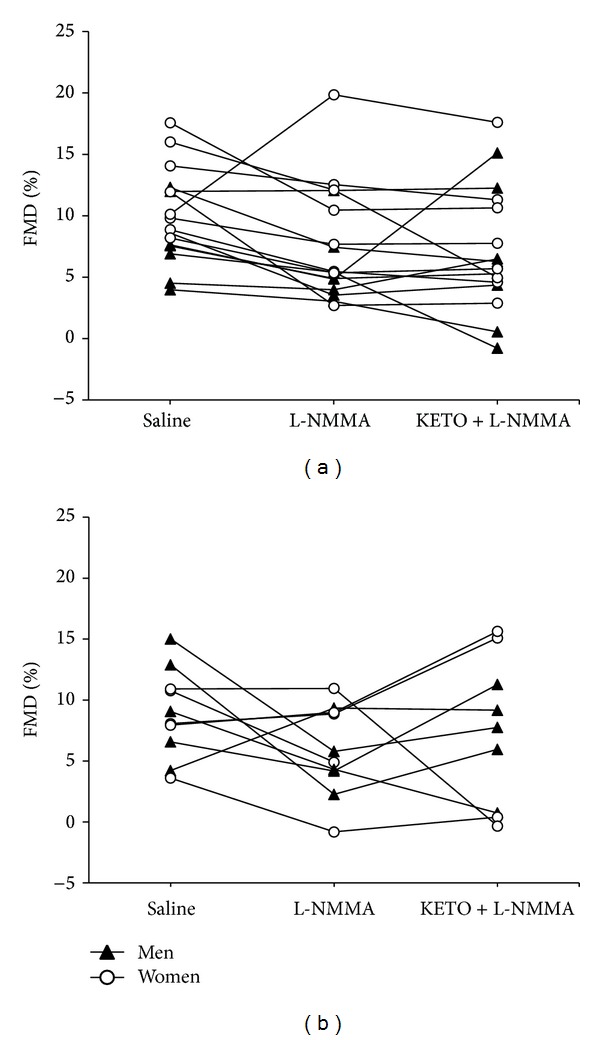
Individual FMD responses to saline, L-NMMA, and KETO + L-NMMA infusion are shown for young (data from young participants have been published previously [8]) (a) and older adults (b). FMD: flow-mediated dilation; L-NMMA: N(G)-monomethyl-L-arginine; KETO: ketorolac.

One female participant in the older group was not included in the analysis between the L-NMMA and ketorolac + L-NMMA trials due to corruption of the video file during data acquisition. In older adults (*n* = 10) L-NMMA infusion decreased RA FMD by 26.5 ± 67.3% compared with saline (8.9 ± 3.6% to 5.9 ± 3.7%), although this was not statistically significant (*P* = 0.08) (Figure 1(a)). Furthermore, the lack of a significant trial ∗ group interaction (*P* = 0.74) indicates that the RA FMD response to L-NMMA infusion did not differ from the decrease (23.7 ± 37.0%) observed previously in young adults (10.0 ± 3.8% to 7.6 ± 4.7%; *P* = 0.03) (Figure 1(a)). Postocclusion AUC SS did not differ between trials or age groups (Figure 1(b)). Normalization to the shear stress stimulus abolished the effect of L-NMMA in both groups (trial ∗ group: *P* = 0.75) (Figure 1(c)). Individual RA FMD responses to L-NMMA in young adults have been reported previously [8] (Figure 2(a)). Compared with saline, RA FMD in older adults was decreased (*n* = 6, range = 36–123% decrease), augmented (*n* = 3, range = 10–122% increase), or unaffected (*n* = 1, 0.4% increase) following L-NMMA infusion (Figure 2(b)).

Relative to L-NMMA, ketorolac + L-NMMA infusion increased RA FMD by 1.0 ± 70.1% (trial: *P* = 0.68) and 52.5 ± 101.3% (trial: *P* = 0.52) for young and older adults, respectively (trial ∗ group: *P* = 0.38) (Figure 1(a)). Shear stimulus normalization did not alter the effect of ketorolac infusion in either age group (trial ∗ group: *P* = 0.56) (Figure 1(c)). Similar to the above, absolute RA FMD responses in older adults were no different (trial: *P* = 0.24) from saline (0.20 ± 0.08 mm) following L-NMMA (0.13 ± 0.09 mm) and ketorolac + L-NMMA infusion (0.14 ± 0.13 mm). Individual RA FMD responses to combined infusion in young adults have been reported previously [8] (Figure 2(a)). Compared with L-NMMA, RA FMD in older adults was decreased (*n* = 2, 83–103% decrease), increased (*n* = 6, 34–170% increase), or unaffected (*n* = 1, 2% decrease) following ketorolac + L-NMMA infusion (Figure 2(b)). Absolute RA FMD responses in young adults were decreased (trial: *P* = 0.01) from saline (0.21 ± 0.08 mm) following L-NMMA (0.15 ± 0.07 mm) and ketorolac + L-NMMA infusion (0.14 ± 0.09 mm). There was no difference between age groups in the number of participants who exhibited decreased RA FMD from saline to L-NMMA (*P* = 0.11) and L-NMMA to ketorolac (*P* = 0.61).

Linear regression, comparing the change in RA FMD from saline to L-NMMA with select independent variables (i.e., age, total cholesterol, MAP, SBP, DBP, and BMI), did not reveal any statistically significant relationships when age groups were combined (all *P* > 0.15 for independent variables). The addition of age group into the model did not influence the results (all *P* > 0.27 for independent variables).

## 4. Discussion

The present findings agree with our previous observation [8] that blocking NO or PN synthesis does not uniformly reduce conduit artery RA FMD in healthy adults. Moreover, in older adults, substantial variability occurred in the magnitude and direction of shear stress-induced vasodilation in response to single and combined blockade of NO and PN synthesis. The present RA data coincide with previous findings in healthy adults [8, 9], including recent data obtained in the brachial artery [10], further questioning the widespread assumption that conduit artery FMD following temporary distal ischemia is primarily NO mediated [2–4, 6].

An age-related decline in FMD has been described in many studies [25–27]. To our knowledge, our study is the first to investigate the impact of healthy aging on the contribution of NO and PN to conduit artery FMD and adds to previous studies investigating the vasodilator pathways contributing to rest and exercise limb hyperemia in aged humans [16–19]. We hypothesized that blocking NO and PN would reduce RA FMD to a greater extent and more uniformly, in older versus younger adults indicating impairment of alternative or redundant vasodilator pathways. However, in contrast to our hypothesis, blocking NO and PN did not consistently reduce RA FMD in older adults. Specifically, only 22% (2 of 9) of older adults exhibited reductions in RA FMD to combined blockade (83–103% decrease). In comparison, 44% (7 of 16) of adults ~40 years younger exhibited reduced RA dilation following ketorolac + L-NMMA infusion (10–115% decrease) [8]. Older adults exhibited substantial variability in the magnitude and direction of RA FMD responses following single and combined blockade of NO and PN suggesting that redundant vasodilator pathways are present in healthy older adults and that aged arteries are able to compensate for loss of specific vasodilators.

We observed that 9 of 16 young and 6 of 9 older adults exhibited an increase in RA FMD in response to combined PN and NO blockade, indicating that non-NO and PN mechanisms are able to compensate for the loss of these two vasodilators and further supporting the existence of redundant vasodilator pathways in humans. Alternatively, our observation of increased FMD in 6 of 9 older adults following combined NO and PN blockade compared to L-NMMA alone might suggest a vasoconstrictor role for PN which is consistent with previous findings [28]. By inhibiting NO and PN synthesis, it is assumed that the remaining dilatory response is attributable to EDHF [29]. However, the involvement of EDHF was not directly tested in our study design. Furthermore, the contribution of additional vasodilators to endothelial responses in healthy adults, including cytochrome P (CYP)450 epoxygenases [30, 31], cannot be ruled out. Infusion of the specific CYP 2C9 inhibitor sulphenazole reduces RA FMD from 11.5% to 7.4% [31], demonstrating a role for CYP 2C metabolites in the FMD response. Single (L-NMMA) and combined (sulphenazole + L-NMMA) blockade lowered RA FMD to 6.0% and 3.9%, respectively [31], suggesting multiple, redundant pathways underlying shear stress-induced dilation. Redundancy occurs in the pathways contributing to the hyperemic response induced by ischemic exercise in young adults [32] suggesting that redundant vasodilator systems are involved in the adequate delivery of blood to metabolically active tissues during exercise. Further support of the importance of redundant vasodilator pathways to cardiovascular function is demonstrated by the maintenance of resting and exercise-induced increases in coronary blood flow following administration of specific inhibitors of vasodilation in healthy men [14]. The relatively robust RA FMD responses observed in healthy adults following NO and/or PN blockade indicate that redundant vasodilator systems, which are likely beneficial for cardiovascular health [12, 14], compensate for the loss of specific vasodilators and that aging in the absence of additional CVD risk factors does not impair these vasodilator systems.

Further research is warranted to determine if redundant vasodilator systems are impaired in clinical conditions associated with endothelial dysfunction. Currently, limited data exist regarding the mechanisms underlying endothelium-dependent dilation in individuals at increased risk for CVD. Patients with essential hypertension [33] and heart failure [29], conditions characterized by the presence of endothelial dysfunction [34, 35], utilize EDHF to maintain endothelium-dependent dilation through activation of the CYP450 epoxygenase pathway. However, EDHF is insufficient in maintaining dilation to levels seen in healthy adults as evidenced by only partial compensation for impaired NO bioavailability by EDHF [33]. Evidence that inhibition of CYP450 epoxygenases does not affect resistance vessel dilation in healthy older adults [36] or conduit artery dilation in patients with essential hypertension [30] suggests that partial compensation to impaired NO bioavailability [33] is dependent on the vascular bed and/or clinical condition studied. Whether the inability of the vascular system to compensate for the loss of specific vasoactive mediators, as observed in certain chronic disease states, underlies future vascular disease remains to be determined.

We acknowledge that our observation of variable responses within individuals to drug infusion may be explained by factors other than the existence of redundant vasodilator pathways, including individual differences in drug sensitivity/metabolism, drug order effects, and the inherent variability of FMD testing. Furthermore, we cannot exclude the possibility that conduit artery FMD of older subjects may be altered by nonendothelium mediated mechanisms. In the present study, sodium nitroprusside was not infused to exclude potential differences in endothelium-independent dilation due to aging [25, 37], a finding not supported by others following administration of exogenous NO donors [36, 38]. We did not perform an additional catheterization visit in older adults to test the effect of drug order on dilator responses due to a lack of statistically significant effects observed previously in young adults who participated in a reverse drug order trial (*n* = 8) [8]. Our existing data [8] support the efficacy of the NO and PN blockade in the present study. For example, we have previously reported that thromboxane B2, an indicator of platelet cyclooxygenase (COX) activity during whole blood clotting, dramatically decreased in a younger cohort following an identical combined infusion protocol [8] demonstrating effective blockade of PN. Furthermore, doses of ketorolac were infused that equaled or exceeded those used previously to block PN [18, 20, 21]. Future studies should investigate whether infusion of a selective COX-2 inhibitor rather than our use of a nonselective COX inhibitor impacts endothelial function differently, an effect observed previously in hypertensives [39]. Infusing acetylcholine would have directly confirmed the efficacy of NOS inhibition. However, our infusion dose of L-NMMA was equivalent or greater than the dose used in previous studies [4, 6, 18, 20–22] and our observed decrease in RA vascular conductance following L-NMMA infusion was similar to previous reports [4, 8, 9, 22, 40–42] supporting that NO synthesis was effectively blocked by L-NMMA infusion in the present investigation. Future infusion studies are needed utilizing a larger number of participants and multiple trials with each drug to better understand the dependence of conduit artery FMD on specific vasodilators in humans and the impact that cardiovascular risk factors have on these vasoregulatory pathways.

## 5. Conclusions

Single and combined blockade of NO and PN synthesis did not consistently reduce RA FMD in older adults adding to the existing literature that NO inhibition does not uniformly decrease conduit artery FMD in healthy humans. Rather, there is substantial variability in the individual RA response to NOS inhibition suggesting a variable contribution of NO to conduit FMD. Therefore, the persistent assumption that conduit FMD is primarily a NO-dependent response limits our interpretation of findings regarding mechanisms both underlying and improving blunted FMD. Furthermore, a large proportion of older adults exhibited increased or minimally reduced RA FMD responses following combined NO and PN blockade suggesting the existence of redundant vasodilator pathways and indicating that aging per se does not uniformly impair these pathways. Future studies are warranted to investigate if the collective influence of additional factors such as disease and/or lifestyle impacts the effect of age on conduit vasodilator pathways.

## Figures and Tables

**Table 1:** Participant characteristics^a,b^.

	Young (*n* = 16)^c^	Older (*n* = 10)
Age (years)*	28 ± 4	65 ± 3
BMI (kg/m^2^)	25.1 ± 2.7	24.2 ± 3.7
SBP (mmHg)*	106 ± 10	119 ± 10
DBP (mmHg)*	65 ± 7	71 ± 6
HR (bpm)	58 ± 9	60 ± 8
LDL cholesterol (mmol/L)	2.4 ± 0.8	2.6 ± 0.5
HDL cholesterol (mmol/L)	1.6 ± 0.4	1.8 ± 0.4
Whole blood viscosity (mPA·s)	5.0 ± 0.7	4.9 ± 0.3
Hemoglobin (mg/dL)	14.2 ± 1.8	14.1 ± 0.8

^a^Data are means ± SD.

^b^BMI: body mass index; DBP: diastolic blood pressure; HDL: high-density lipoprotein; HR: heart rate; LDL: low-density lipoprotein; SBP: systolic blood pressure.

^c^Data from young participants have been published previously [8].

*Significant difference between age groups (*P* < 0.05).

**Table 2:** Baseline hemodynamics across infusion trials in young and older adults^a,b^.

	Saline	L-NMMA	KETO + L-NMMA	Trial
Young (*n* = 16)^c^				
RA diameter (mm)	2.09 ± 0.35	2.08 ± 0.34	2.08 ± 0.36	0.68
RBF (mL/min)	16.4 ± 14.2	11.7 ± 7.5	11.7 ± 5.4	0.05
MAP (mmHg)	77.5 ± 9.9	80.6 ± 11.6	84.0 ± 12.6*	0.01
RVC (mL/min/100 mmHg)	20.9 ± 17.7	14.5 ± 9.6*	14.0 ± 6.3*	0.03
Older (*n* = 10)				
RA diameter (mm)	2.16 ± 0.42	2.12 ± 0.36	2.05 ± 0.46	0.32
RBF (mL/min)	18.0 ± 5.9	13.2 ± 4.0	11.9 ± 6.1	0.01
MAP (mmHg)	82.7 ± 9.4	87.9 ± 11.0	90.3 ± 10.2*	0.01
RVC (mL/min/100 mmHg)	22.9 ± 8.9	15.1 ± 4.4*	13.3 ± 6.9*	0.01

^a^Data are means ± SD.

^b^KETO: ketorolac; L-NMMA: N(G)-monomethyl-L-arginine; MAP: mean arterial pressure; RA: radial artery; RBF: radial artery blood flow; RVC: radial artery vascular conductance.

^c^Data from young participants have been published previously [8].

*Significantly different from saline trial (*P* < 0.05). Trial = *P* value for trial effect for each variable.
